# Differences in the Social Motivations and Emotions of Humans and Other Great Apes

**DOI:** 10.1007/s12110-023-09464-0

**Published:** 2023-11-16

**Authors:** Michael Tomasello

**Affiliations:** 1 Duke University, Durham, NC USA; 2 Max Planck Institute for Evolutionary Anthropology, Leipzig, Germany

**Keywords:** Motivations, Emotions, Human Evolution, Cooperation, Culture

## Abstract

Humans share with other mammals and primates many social motivations and emotions, but they are also much more cooperative than even their closest primate relatives. Here I review recent comparative experiments and analyses that illustrate humans’ species-typical social motivations and emotions for cooperation in comparison with those of other great apes. These may be classified most generally as (i) ‘you > me’ (e.g., prosocial sympathy, informative and pedagogical motives in communication); (ii) ‘you = me’ (e.g., feelings of mutual respect, fairness, resentment); (iii) ‘we > me’ (e.g., feelings of obligation and guilt); and (iv) ‘WE (in the group) > me’ (e.g., in-group loyalty and conformity to norms, shame, and many in-group biases). The existence of these species-typical and species-universal motivations and emotions provides compelling evidence for the importance of cooperative activities in the human species.

When an environmental challenge is urgent and consistently predicted by a particular situation, organisms may evolve reflexes and other kinds of inflexible stimulus-driven responses. Thus, humans have an eye-blink reflex when small objects rapidly approach their eyes and a gag reflex when food obstructs their windpipe. On the other hand, many behaviors of many organisms are produced quite flexibly, with the individual agent making the decision of whether and how to produce them (Tomasello, 2022a). In such cases, natural selection often results in motivations and emotions that bias the individual toward making certain decisions but only if circumstances permit. For example, humans prefer some foods over others, but only if they are obtainable without excessive risks and/or costs.

Mammals have evolved to be flexibly social, and psychobiological research has identified some of the physiological mechanisms that underlie some of their most basic social motivations and emotions, for instance, those involving the hormones oxytocin and vasopressin. Humans share most of these processes with other mammals, but humans also behave socially in some special ways—most especially in the ways they engage with one another cooperatively—and so we might expect to see some species-specific social motivations, emotions, preferences, and attitudes.

My goal in this paper is to review recent research comparing the social behavior of humans to that of their nearest primate relatives to see what kinds of motivational and emotional differences can be discerned. I summarize the most general differences of social behavior between humans and other apes, and in this context review recent empirical studies that suggest the specific motivations and emotions that may underlie those differences. I do this first for social motivations and emotions underlying interactions and relationships among individuals and then for social motivations and emotions underlying life in a cultural group.

## Interactions among Individuals

Humans share with other great apes many of the same individual motivations and emotions for obtaining and consuming food, for mating, and for avoiding predators and other dangers. They also share many of the same social motivations and emotions for such things as staying in the social group, taking care of children, retaliating against aggressors, and many others. But humans are in many ways more cooperative than other great apes. This can be clearly seen in three of the most prominent and important cooperative behaviors in the human species: collaboration, prosocial behavior, and communication. All three of these have associated with them species-typical motivations and emotions.

### Collaboration

Humans’ nearest great ape relatives, as well as many other primates and mammals, form coalitions and alliances in dominance contests, and some hunt small prey in groups. But experimental studies suggest that their motivation to collaborate is purely instrumental: they are using their partners as social tools to obtain a personal benefit. In contrast, human children’s proximate motivations are not just about providing self-serving benefits but rather about providing mutualistic benefits. This pattern may be seen in several different types of experimental comparisons.^1^

Most directly, human children have a **stronger motivation to collaborate** with others to obtain food than do other apes. Thus, when chimpanzees were given the choice of obtaining food for both themselves and a conspecific either by acting solo or by collaborating with that conspecific, they were indifferent to the choice (since the food was the same in both cases). In contrast, young human children overwhelmingly chose to obtain the food by collaboration (Rekers et al., 2011). A similar motivational difference can be seen in chimpanzees’ and human children’s tendency to persist in collaboration in the face of temptations to defect: chimpanzees cease to collaborate as soon as they have obtained their reward, whereas young children persist until the partner has obtained hers as well (Hamann et al., 2010; Greenberg et al., 2010). Finally, chimpanzees can collaborate with humans to achieve instrumental goals, but when the collaboration is simply a game with no reward, they are uninterested, whereas children are equally interested in both types of collaboration (Warneken et al., 2006). (NB: MacLean & Hare, 2013, found that chimpanzees and bonobos did engage in some cooperative games with humans, although they did not do so with conspecifics.)

Because depending on collaborative partners is always risky, humans have evolved the motivation to form with others a joint commitment to collaborate. Joint commitments create species-typical **normative motivations or obligations**. Human children at some point begin overtly inviting a partner to collaborate and wait until the partner agrees to the invitation before initiating the joint action. If the partner does not live up to their joint commitment, children normatively protest (e.g., “You can’t do it like that. You have to do it this way.”), using the normative language of *must, should*, and *ought*. Moral philosophers from the time of Smith (1759/1982) have dubbed the emotion elicited by a partner reneging on a commitment as **resentment** for not being treated as an equally deserving and respected partner. The normative force of joint commitments is also clear when children overtly forewarn others when they themselves must (for a good reason) break that commitment (e.g., Gräfenhain et al., 2009), and they expect others to do the same (Kachel et al., 2019). In addition, young children often apologize after breaking commitments, and even go to special pains to repair any damage done, suggesting an emotion of **guilt** (Vaish et al., 2016).

Other great apes do not seem to make joint commitments. In situations where collaboration might be risky, chimpanzees nevertheless take off toward the potential collaborative goal and just hope the other will follow, with no initiating communication (Duguid et al., 2014). Heesen et al. (2020) have recently observed that when bonobos are mutually grooming and one of them is pulled away by the lure of food, after the food has been consumed the individual more often returns to the mutual grooming than to any other activity—which they believe is evidence for a joint commitment. But this just shows that the bonobo enjoys mutual grooming more than other activities, not a joint commitment to the activity (Tomasello, 2022b). Children’s joint commitments are often made in language, and so we would not expect explicit joint commitments in great apes. But in some situations (i.e., when both potential partners’ motivation to collaborate is obvious to both) all that is needed for human children is a “knowing look” between potential partners, or else a simple gesture like pointing to the potential collaborative goal. Both of these behaviors are, in principle, available to chimpanzees, but they do not use them, whereas young children use both of these nonverbal methods to establish a joint commitment to collaborate (Siposova et al., 2018). Likewise, nonhuman great apes do not have the linguistic abilities for human-like normative protest and apologies; but still, when one partner stops collaborating we do not see nonhuman great apes becoming inordinately upset, and if they themselves stop collaborating, they do not behave in any special way toward their former partner.

And so it seems clear that humans have evolved an especially strong motivation to collaborate, including even a sense of obligation to one’s partner (which they feel guilt for breaking), and they expect such a motivation and sense of commitment from their partner in return (whose breach they meet with expressions of resentment).

### Prosocial Behavior: Sharing

Humans also seem to have evolved some new motivations for sharing the spoils of collaborative activities. The most general observation is that when nonhuman great apes have food in their possession, they are mostly reluctant to share it with others, whereas young children, while also reluctant to some degree, share more generously. (Although bonobos may be somewhat more generous than other apes [Tan et al., 2017], this seems to depend on the cost of sharing being low; see Bullinger et al., 2013.)

A special situation for resource sharing is after a collaboration. Chimpanzees share food with others after a group has captured a monkey, but they do so only under pressure from those who are begging and harassing them (Gilby, 2006), or else they share with coalition partners for instrumental purposes (Mitani & Watts, 2001). Young children share resources with one another after a collaboration much more freely, without any pressure from a partner or any other instrumental motivations (Warneken et al., 2012). The difference comes out clearly in comparative experiments. First, Hamann et al. (2012) had pairs of children and pairs of chimpanzees collaborate to obtain food, but then most of the food ended up in the possession of one partner. The children, but not the chimpanzees, then shared some of their excess bounty with the partner (i.e., just enough to equalize), which the chimpanzees did not do. Ulber and Tomasello (2017) tried to make it easier for both children and chimpanzees, but still found the same result. Melis et al. (2011) compared chimpanzees’ food sharing with individuals who had acted as collaborative partners and those who were just free riders and found no difference in the sharing—whereas Melis et al. (2013) found that children rewarded collaborators more than free riders. Combined with findings that young children also give more than equal resources to a partner who worked more than they did to obtain those resources (Hamann et al., 2014), the conclusion is that human children, but not chimpanzees, have a sense of the **deservingness** of their partner when sharing food with them. Likewise, when their partner does not share with them to the degree that they feel they deserve, they normatively protest (Rakoczy et al., 2016).

In other words, children but not chimpanzees are motivated to treat collaborative partners with **fairness**. Well-known studies by de Waal and colleagues have been used to suggest that capuchin monkeys and chimpanzees have a sense of fairness as well (i.e., they object to being treated unfairly). But six different laboratories have failed to replicate the Brosnan and de Waal (2003) experiment with capuchin monkeys when more stringent control conditions are employed (see Tomasello, 2016, for specific citations), and three studies from a single laboratory have failed to replicate Brosnan et al.’s (2005) experiments with chimpanzees when more stringent control conditions were employed (see Engelmann et al., 2017).

Engelmann and Tomasello (2019) hypothesize that humans’ sense of fairness is based on **mutual respect** among collaborative partners. Collaborative partners are both causally necessary in producing the resources, and their understanding of how the roles in the collaboration must be played involve standards that apply to everyone equally in that role (partners even could reverse roles). The straightforward conclusion is thus that equal partners should share equally (with perhaps some consideration of merit as well). Support for this ‘fairness as equal respect’ hypothesis comes out most clearly in the phenomenon of procedural fairness. Young children are content with receiving fewer rewards than other partners in a collaboration if (and only if) the procedure by which the rewards were distributed was impartial (e.g., rolling dice) or else they have been shown respect as equal partners in some other way (Grocke et al., 2015, 2018). The phenomenon of procedural fairness has not been studied, to my knowledge, in great apes, but most observers, I would venture to guess, would be surprised if chimpanzees took into account how the distribution was effected.

And so again it is clear that humans have evolved some special motivations around the sharing of resources. Gurven (2004) documents quantitatively that food sharing is ubiquitous and frequent across human societies in a way that it is not in the societies of other great apes. This seems to be based on especially human social attitudes and motivations such as deservingness, mutual respect, and fairness.

### Prosocial Behavior: Helping

In social environments dominated by competition for resources, it is important to make and keep “friends” that can be allies in dominance contests. Many studies have shown that chimpanzees and bonobos form coalitions, share food, and groom preferentially with selected individuals that may be called friends. As noted above, after a successful group hunt chimpanzee individuals tend to share meat preferentially with their previously established friends. It is in this context that Mueller and Mitani (2005:278) observe: “Competition … frequently represents the driving force behind chimpanzee cooperation.”

In experiments, chimpanzees and bonobos help both friends and non-friends (e.g., to reach a desired but out-of-reach object) fairly readily and without any immediate reward—as long as the effort and/or costs for doing so are not high (see Melis, 2018, for a review). One hypothesis is that when they are helping non-friends they are, in essence, offering friendship. Thus, in experiments, when a chimpanzee is helped by another in some way, she is more likely to provide help in return than in control conditions when no original help is provided (e.g., Schmelz et al., 2017). Knowing that this is how things work, individuals may then help others in anticipation of help in some future situation. It is not that individuals are keeping score quantitatively, but rather they are engaged in what de Waal (2000) calls attitudinal or emotional reciprocity. They are forming friendships based on the positive affect generated both to those who provide one with help and to those who receive one’s help. This is likely based on the same psychological mechanism by means of which youngsters identify and become bonded to their mothers or other caregivers: youngsters come to have positive affect toward anyone who is continually helping them. It is noteworthy that chimpanzees have more oxytocin in their systems immediately after they have either shared food or received food from another individual (Crockford et al., 2013).

Human collaboration provides another basis for helping, namely, interdependence. To the degree that individuals are interdependent with one another it is in their evolutionary interest to help one another (Roberts, 2005; Tomasello et al., 2012). The specific hypothesis is that humans, like other great apes, have a motivation to help other individuals as a way of either initiating or maintaining positive affective bonds of friendship. But, in addition, humans added to this personal motivation an intrinsic motivation just to see that others in the group received the help they needed no matter who provided that help. This may be seen as a motivation of **intrinsic sympathy**. Thus, in an experiment, young children witnessed an adult needing help, and this created arousal (the dilation of their pupils). They were equally satisfied (their arousal returned to baseline level) both when they helped that person and when they saw her being helped by a third party (and more satisfied in both of these conditions than when the person was not being helped at all). In contrast, Hepach et al. (2021) found that, unlike the children, chimpanzees were more satisfied when they provided the help to a conspecific themselves than when they saw a third party doing it. This is presumably because at least part of their proximate motivation or goal was to receive “credit” for providing that help—whereas the children’s proximate motivation or goal was simply to make sure that the other person was helped (based on a general propensity to monitor the welfare of those on whom one depends).

Similar differences between humans and other great apes are apparent in a related experimental paradigm. The phenomenon is paternalistic helping. If a human sees an individual needing a certain kind of help (e.g., with injuries), they will provide that help even if the individual is asking for something different. In a comparative experiment, both chimpanzees and human children saw a conspecific reach for one of two tools. In the key condition, he reached for a tool that the subject knew was dysfunctional; it was not the one that the helpee actually needed. Chimpanzees nevertheless fetched for him the one for which he was reaching. In contrast, three-year-old children helped an adult paternalistically, fetching for him the tool they knew he actually needed. These results are consistent again with the hypothesis that the evolutionary function of chimpanzees’ helping behavior is based on the return benefits anticipated (friendship and its benefits), whereas the evolutionary function of children’s helping behavior is to make sure that others in the group are getting whatever help they need. For chimpanzees, the social bond is strengthened if they give the helpee what she wants, but human children can afford to have others provide the help instead of them—so long as the helpee is helped—and to assist others paternalistically even if it means temporarily denying them what they want.

And so once again humans and other great apes behave in generally similar ways but differently based on different proximate motivations. Apes and humans both help in order to initiate and maintain friendships—which requires providing requested help oneself—whereas humans also help just to make sure that the other person receives the help she needs, which just requires making sure that that takes place, no matter who provides the help and no matter what the recipient wants. We may thus say that humans evolved an intrinsic motivation for helping in which the main goal that the recipient gets the help she needs based on intrinsic sympathy.

### Communication

The communication of most animal species is aimed mainly at getting others to do what one wants them to do; it is imperative communication. Great apes communicate almost totally in this way in both the gestural and vocal modality (with a few rare exceptions; see below). Humans communicate for this goal as well, but they also have other, more cooperative communicative goals.

Humans communicate with others not only to request things but also for two other motives. First, they **inform** others of things helpfully. Thus, when faced with an adult searching for something, even prelinguistic infants will helpfully inform her where that thing is with no apparent reward for herself (e.g., Liszkowski et al., 2006, 2008). The difference to great apes comes out clearly in a comparative experiment (Bullinger at el., 2011). Both young children and great apes were faced with the opportunity to point for a hidden tool. In one condition it was clear that the tool would be used to retrieve a reward for the pointing subject (so the pointing was selfish or “for-me”), whereas in the other condition it was clear that the tool would be used to retrieve the reward for the experimenter (so the pointing was helpful or “for-you”). The chimpanzees pointed reliably only when they themselves benefited, whereas the human children pointed reliably no matter who benefited. There is one context in the wild in which chimpanzees may inform others of things helpfully by warning them of a specific danger (Crockford et al., 2012), but this seems to be a very rare context, whereas informative communication is ubiquitous and frequent in all human societies.

Second, humans communicate with others simply to share experience. Thus, when prelinguistic infants point enthusiastically to something, they are only satisfied if the adult responds by both identifying the intended referent and by responding enthusiastically in turn (Liszkowski et al., 2004). The infant’s goal seems to be to share an emotion or attitude with the adult about some referent, a behavior which may have precursors in the way that infants engage in various forms of “emotional attunement” with caretakers from early in development (Stern, 1985). Moreover, later in ontogeny, adults spend much of their time simply gossiping or “passing the time of day” with others. This presumably indicates a **motivation to share experience** just for its own sake (also apparent in various modern social media).

Again, humans share many aspects of their communicative activities with other great apes, but again they have evolved motivations, in this case communicative motivations, that are unique among great apes in being aimed at one or another form of cooperation: helping by informing or simply sharing experience as a unique form of social interaction.

### Summary

Tomasello (2016) provides a categorization scheme of different forms of human cooperation, including morality, that helps unify these prototypically human social motivations. First are motivations that may be characterized as **‘you > me’**, basically prosocial motives in which the individual places the interests of another individual in front of her own. This is characteristic of the social motivations we have reviewed here of helping and informative communication based on prosocial sympathy. Second are motivations that may be characterized as **‘you = me’**, for example, the sense of mutual respect and deservingness leading to a propensity to treat others fairly. And third are motivations that may be characterized as **‘we > me’**, basically valuing the collaborative motives over individual motives, for example, the sharing of experience and the feelings of commitment, resentment, and guilt in which the concerns of our shared agency take precedence over my individual preferences and desires. The evolutionary hypothesis is that at some point in their evolution humans came to cooperate with others in foraging, in parental care, and other activities—thus creating a much stronger and more urgent web of interdependencies—and this led to a whole host of more cooperative social motivations, emotions, and attitudes than had previously been characteristic of the species (Sterelny, 2012; Tomasello, 2016).

## Interactions with the Group

Early humans lived in social groups, perhaps similar to those of modern-day chimpanzees and bonobos. This meant that they were personally familiar with most individuals in their group, and, if not, they could identify them as group members by their continuing physical proximity in the same territory. Strangers, especially on the margins of the group’s territory, would have been treated with suspicion and/or aggression. Chimpanzees actually engage in so-called border patrols to guard against encroachment by neighboring groups, and sometimes even attack neighbors. Even early humans would have feared being separated from the group and would have felt a motivation to stay in physical proximity to the group (perhaps based on the same motivation leading youngsters to stay near their mothers early in life).

But at some point, at the very latest with modern humans, a subtle but important difference emerged. Perhaps because their collaborative life was so successful, human groups began to increase in population to the point where they began to split off into subgroups. Nevertheless, if it came down to competition or combat with rival groups, these subgroups still considered themselves all part of the same cultural group (this is often called tribal organization). But now the individual’s group included in-group strangers, many of whom lived in different territories, which created the need for individuals to recognize groupmates in ways not based on either personal familiarity or common territory. The new basis for recognition was similarity, initially of behavioral practices, since modern humans already employed powerful means of cultural transmission for tool use, food preparation, linguistic conventions, etc. At some point, physical appearance, including adornments, also became important identifiers.

### Group Identity: Conformity and Common Ground

In this context, it was important for modern human individuals to make sure that others could easily identify them as members of the group as well, so that they would know that (i) we are on the same team if it comes to competition or combat with other groups, and (ii) we are familiar the same cultural conventions and so can readily communicate and coordinate in collaborative activities. This concern for identification with the group meant that modern humans evolved an especially strong **motivation to conform** to the behavior of others in their group. This included appearance, and indeed even young children understand themselves to be members of the same group if they are simply wearing the same color t-shirt and hat as other children (experimentally constructed “minimal group”; Dunham, 2018).

Importantly, the new motivation was not just to do what others were doing for instrumental purposes but to conform to their behavior even when there were no instrumental advantages for doing so. For example, Haun and Tomasello (2014) had great apes and 2-year-old children learn on their own how to operate an apparatus which ejected a reward. Then, they saw three conspecifics operate it differently, and the apparatus still ejected a reward. When given the opportunity to operate the apparatus again, the apes ignored the demonstrators and stuck with the method that had worked for them in the past. In contrast, most children switched methods; they conformed to the group, even if he had had no advantages over their previously successful strategy (see also Horner & Whiten, 2005). Similarly, Lyons et al. (2007) had young children observe an adult perform clearly causally irrelevant actions, but the children often reproduced those actions nonetheless, even though they knew (as determined by a later interview) that the actions were causally irrelevant; bonobos again do not engage in such so-called overimitation (Clay & Tennie, 2017). Importantly, a number of studies show that young children conform even more strongly to members of their in-group than to members of some out-group (see Over & Carpenter, 2012, for a review). Based on these and other studies, Keupp et al. (2013) concluded that children’s strong conformity to their group is based on their kind of normative inference—this is how “we” do things—that is in many cases strong enough to override instrumental concerns. Group conformity is based on group identity, which may be most clearly seen in adults’ and children’s conformity to various cultural rituals, presumably in order to display a costly signal of group identification (Wilks et al., 2016).

In addition to similarity in behavior, also important to the formation of a cohesive cultural group is similarity of experience, both direct and indirect through communication, as noted above. But individuals in a cultural group also have the **motivation to share experience through narratives**, that is, experiences for which their interlocutor was not physically present. Important are not only narratives in which individuals relate stories of personal experiences but also culturally shared narratives about such things as the origins of our people. In the telling of narratives, individuals share not only experiences but also, in the moment, the expression of attitudes about those events (e.g., disappointment that a hunt had proved unsuccessful). In general, communication in a conventional language and in conventional narrative form expands the common ground of the members of a cultural group.

So beyond great apes’ social learning, modern humans evolved a strong motivation to conform to the behavior of others in their group, independent of any instrumental consequences. They also expanded the ways in which they shared experience and so built up common ground with one another communicatively. In both cases, the individuals within a social group became more similar to one another in behavior and experience, and this increased their identification and affective bonds with their compatriots, including in-group strangers.

### Group Identity: Social Norms

It is in the interest of members of a cultural group that others in their group conform to the groups’ practices as well, both to facilitate coordination and to clearly mark them as group members. Thus arose group-wide expectations for individual behavior, a.k.a. social norms, mostly enforced via threats to reputation (and potentially to group membership). Social norms very likely arose with modern humans, as apes show no signs of group-level social norms (although they engage so-called policing behavior, it is mainly aimed at breaking up fights and diffusing tense situations to the benefit of the policer who stands to be adversely affected; Tomasello, 2016).

From around 3 years of age, young children begin to display a **motivation to enforce social norms** on others. Thus, when children of this age detect a social norm violation, they protest, often normatively, and importantly, they do this even when they themselves are not participating in the relevant activity and so are not directly affected by the norm violation (see Schmidt & Tomasello, 2012, for a review). The children are presumably motivated by something like a concern for how things are going in the group in general, as indicated by their use of impersonal normative language concerning how “we” do it or should do it (or even “how it is done”). This language makes it clear that children are not just expressing their personal preference or desire, but rather they are referencing the group’s normative standards that apply to all group members alike. If the norms are just about conformity (conventions), children enforce them selectively only on in-group members (Schmidt et al., 2012). Children are not enforcing social norms directly for their own individual personal benefit; they are enforcing social norms to facilitate the smooth functioning of the group as such.

Further evidence for this interpretation comes from the fact that when young children make up social norms or rules themselves, mostly in games, when it is time to teach the game to others they enforce these self-invented norms on the novices using normative language (e.g., “You have to play it this way”). And when children observe someone participating in the creation of a norm but then breaking it, they protest normatively even though this does not affect them at all (Schmidt et al., 2016). The ways that young children understand and deal with the norms that they have created themselves suggests further that their concerns are not with their own well-being, but rather with the smooth functioning of the group. This suggests a kind of collective commitment social norms that is a kind of scaled-up, group-level version of their joint commitments with collaborative partners: this is how “we” do things, and for the good of the group you should do them in this way also. When members act against the interest of the group, other members often feel **moral outrage or indignation** (a kind of group-level resentment) that they could endanger the group in that way.

As with conformity, following and enforcing social norms identifies one with the group and a commitment to its ways of doing things. Given the recognition that others in one’s cultural group will have normative expectations about one’s actions—and this will have powerful repercussions on one’s status in the group—individuals developed the **motivation to manage their reputation** in the group in a manner not characteristic of other great apes (and so, perhaps, the earliest humans). In a comparative experiment, young children were more motivated to share with others and less motivated to steal from others when someone was watching, especially if that other person was from their own cultural (minimal) group (Engelmann et al., 2012, 2013)—but chimpanzees did not care either way if a conspecific was watching or not.

Also associated with reputation management in the group are special emotional displays designed to forestall the norm enforcement and negative reputational judgments of others. Thus, when individuals are not conforming to the expectations of others in the group, and those others find out, they often display emotions such as **shame and embarrassment.** And these displays work. Even young children are more positive towards a transgressor (e.g., one who has been careless and so damaged a valued toy) if that transgressor outwardly displays some kind of conciliatory emotions such as shame, guilt, embarrassment, remorse, or even offers an apology (Vaish et al., 2016). Such displays convey that even though one has transgressed, one has respect for the victim and shares her social norms and values, and so should not be inordinately sanctioned or excluded from the group. The negative valence of these emotions themselves serve to reduce the agent’s tendency to transgress, and the emotional displays serve to mitigate the social consequences of any transgressions committed.

### Group Identity: In-Group Favoritism

Because of their greater familiarity and positive social bonds with others in their cultural group (as opposed to members of out-groups), humans have developed a special sense of **in-group trust**. They trusted that others in their group would treat them cooperatively because everyone in the group depended on everyone else. They themselves, in turn, showed **loyalty to the group**. For example, young children who have been experimentally assigned to a minimal group (same color t-shirts, etc.) show loyalty by preferring to stick with the group even when it means losing rather than winning a game (Misch et al., 2014). In general, preschool children begin to show an understanding of the group as a kind of collective agency (Noyes & Dunham, 2017).

Loyalty to the group also translates into **in-group favoritism**, that is, a motivation to benefit members of one group over out-group members, as well as to look out for the welfare of the group as such (over other groups). Thus, preschool children preferentially help in-group over out-group members (Over, 2018), and they share resources with children in their in-group more generously than with children from an out-group (Fehr et al., 2008). Moreover, when the issue of their minimally established in-group’s reputation is at stake, young children engage in active attempts to manage other people’s evaluative judgments of their in-group as a whole (Engelmann et al., 2018). Moreover, when a member of a child’s minimally established in-group does something mean to a victim, she feels an in-group responsibility to make amends to that victim—**collective guilt**—whereas she does not feel a responsibility if the perpetrator was an out-group member (Over et al., 2016). One can speculate that these various forms of in-group favoritism (and out-group disfavoritism) are basically a scaled-up version of children’s tendency to favor collaborative partners over non-collaborators (i.e., free riders) in more direct cooperative activities.

Another expression of humans’ prosociality toward group members is their **motivation for pedagogy**. Adults engage in pedagogy as a kind of prosocial act: they sacrifice some time and effort to make sure that a child or other person learns something that will be useful to her. Like other forms of informative and narrative communication, pedagogy also serves to increase the common ground among the teacher, the learner, and everyone else in the group, since most forms of pedagogy are expressed as culturally conventional information or skills: “this is how we do it” or “this is a fact about the world that we have established.” I know of no empirical data, but presumably adults prefer to teach children in their own in-group over other children (indeed, pedagogy very likely first evolved with one’s own offspring).

In all, modern humans are characterized by a group-minded psychology, and this is one of the most firmly established findings in all of social psychology (for research with young children see the reviews of Dunham et al., 2008, Dunham, 2018). They show their in-group favoritism and trust in many ways that is not characteristic of other great apes, and so presumably it has emerged in more or less recent human evolution.

### Summary

Above I summarized and categorized human’s species-typical cooperative motivations in interacting with other individuals as: you > me, you = me, and we > me. In the case of human’s species-typical cooperative motivations in interacting with the group, we can widen our scheme a bit and posit three other forms. First, humans have a tendency to identify with their in-group we (which I will indicate as WE), in a pattern we may designate as WE = me, meaning that I see myself as a member of the group. This comes out especially in individuals’ motivation to conform to the group and to share experience with them. Second, humans have a tendency to favor the group over themselves in some instances to some degree: WE > me. This comes out most clearly in their motivation to manage their reputation so as to ensure that they are approved by the group. It also comes out in their motivation to enforce social norms on others and engage in pedagogy with others as a benefit to the group’s functioning as such (and in both of these activities the norm enforcer or pedagogue actually purports to be speaking for the group not herself as an individual), and to engage in other ways morally with others in the moral community. Third and finally, in many of these behaviors there is a favoritism of the in group over the out group, which may be summarized in the formula WE > they. This comes out in human favoritism for in-group over out-group in helping and sharing, as well as in various sacrifices for in-group (loyalty, trust, collective guilt) that are not shown to out-groups. The overall conclusion is that at some point modern humans came to identify with and cooperate in selective and especially powerful ways with others in their cultural groups—as kind of scaled up collaborative groups—and this led to a whole host of more cooperative social motivations, emotions, preferences, and attitudes than had previously been characteristic of the species.

## The Evolution of Human Sociality

Humans engage in many of the same basic activities, underlain by many of the same basic motivations and emotions, as other great apes. For example, all great apes, including humans, have to find food and consume it, find mates and mate, identify predators and escape, and cultivate “friends” as allies and supporters. And so it is to be expected that these sister species have many of the same basic motivations and emotions, including not only such basic things as hunger and sexual desire, but also the “basic emotions” of fear, anger, happiness, sadness, and surprise.

But other aspects of great apes’ social lives are very different. As a somewhat solitary species, orangutans have less motivation than other great apes to do everything in a group; as a less food-competitive species, bonobos are less hostile to members of neighboring groups than are other great apes; as a species that lives in harems, gorillas are less in need of friends and allies for dominance contests. Humans, by all appearances, are distinguished from other great apes by the inordinate amount of cooperation they display in a variety of activities, especially in foraging, in childcare, and in communication and cultural transmission via pedagogy (Sterelny, 2012). All of these ultra-cooperative activities must be energized and motivated, and the attempt here has been to identify and categorize the energizing and motivating forces. These include both social motivations and emotions aimed at other individuals, as (potential) cooperative partners, and those aimed at the group as a whole or other individuals as in-group versus out-group members. In some accounts, these two actually emerged in this order in evolution, with human species-typical collaborative activities emerging before their species-typical cultural organization (Tomasello et al., 2012). Following the discussion in the text, Table 1 breaks down various categories within this overall dichotomy. The basis of this further categorization is more specificity in the nature of the relationship between the individual (‘me’) and, on the one hand, other individuals (‘you’), and, on the other hand, the partnership (‘we’) or the cultural group ‘WE’.

**Table 1:** Summary of the most important of humans’ species-typical motivations and emotions for cooperative interactions with others and the group. ‘We’ refers to a shared agency with a collaborative partner. ‘WE’ refers to shared agency with compatriots in a cultural group

Other Individuals	In-Group ‘WE’
You > me prosocial sympathy [prosocial helping, communication]	WE = me in-group identity [conformity, sharing experience]
You = me respectful equality [mutual respect, fairness, deservingness]	WE > me reputation & morality [concern for reputation, enforce social norms, pedagogy, shame, indignation]
We > me Normativity [commitment, resentment, and guilt]	WE > they in-group favoritism [parochial helping & sharing; parochial loyalty, trust, collective guilt]

If we view motivations and emotions as natural selection’s way of “putting its finger on the scale” of the individual agent’s decision making for some benefit, it is important to emphasize that different motivations and emotions may be at work simultaneously. For example, individuals respecting others and wanting them to get what they deserve is only one motivation and it can be overcome by other motivations, not only individual self-serving motivations (e.g., to maximize food intake), but also by other social motivations that may encourage a different decision (e.g., to benefit a particular individual over others or to manage one’s reputation in the group). This point highlights the complexity of human decision making and the possibility of sociomoral dilemmas in which different cooperative motives point in different directions. Evolution goes step by step and does not always harmonize each new step with what already exists.

## Conclusion

The challenge of social life for the individual is to balance one’s self-serving needs and goals, which often put one in competition with others, and one’s interdependent needs and goals with others and the group, which requires cooperation. As excessive altruism can be detrimental or lethal for the individual, the trick is to motivate cooperation to just the right degree, by evolving motivations and emotions that bias the individual’s decision-making in a cooperative direction, but still leave it up to that decision-making agent precisely how to balance that motivation for cooperation with various individual needs and goals.

Humans are dependent on others and the group—actually, interdependent with others in the group—to an inordinate degree among primates, and so it is to be expected that they would evolve some distinctive and especially strong motivations and emotions for their various species-typical forms of cooperative activity. Examination of the emotional and motivational psychology of the human species thus provides additional support—that is, beyond just observations of their cognition and behavior—for the hypothesis that the driving force in the evolution of human forms of cognition and sociality is their special forms of cooperation, including both collaboration with individuals and participation in cultural groups.

## Data Availability

Not applicable.

## Footnotes

1 In most cited experiments the comparison is between adult great apes and human children of various ages. For both practical and theoretical reasons, this is the way most studies in the field have been conducted. See Boesch (2007) for a critique of the method and Tomasello and Call (2008) for a defense.
